# Supplementary material for: Supply chain product quality control strategy in three types of distribution channels
Source: PLoS One. 2020 Apr 22;15(4):e0231699. doi: 10.1371/journal.pone.0231699 (PMC7176101; doi:10.1371/journal.pone.0231699)
Supplement: Attachment — Submitted filename: Reviewer 1 Comments.pdf [file pone.0231699.s002.pdf]

Cancel Save and Close

PONE-D-19-20228
"Supply Chain Product Quality Control Strategy in Three Types of Distribution Channels"
Original Submission

Reviewer #1:

**Reviewer Recommendation Term:**	Major Revision
**Rate Review:**	0

Custom Review Question(s):	Response
**Comments to the Author**<br><br>1. Is the manuscript technically sound, and do the data support the conclusions?<br><br>The manuscript must describe a technically sound piece of scientific research with data that supports the conclusions. Experiments must have been conducted rigorously, with appropriate controls, replication, and sample sizes. The conclusions must be drawn appropriately based on the data presented.	Partly
2. Has the statistical analysis been performed appropriately and rigorously?	Yes
3. Have the authors made all data underlying the findings in their manuscript fully available?<br><br>The PLOS Data policy requires authors to make all data underlying the findings described in their manuscript fully available without restriction, with rare exception (please refer to the Data Availability Statement in the manuscript PDF file). The data should be provided as part of the manuscript or its supporting information, or deposited to a public repository. For example, in addition to summary statistics, the data points behind means, medians and variance measures should be available. If there are restrictions on publicly sharing data—e.g. participant privacy or use of data from a third party—those must be specified.	Yes
4. Is the manuscript presented in an intelligible fashion and written in standard English?<br><br>PLOS ONE does not copyedit accepted manuscripts, so the language in submitted articles must be clear, correct, and unambiguous. Any typographical or grammatical errors should be corrected at revision, so please note any specific errors here.	No
5. Review Comments to the Author<br><br>Please use the space provided to explain your answers to the questions above. You may also include additional comments for the author, including concerns about dual publication, research ethics, or publication ethics. (Please upload your review as an attachment if it exceeds 20,000 characters)	1. The language of the article should be more concise and accurate.<br>2. It is better to complement the differences between this study and the existing research literature.<br>3. The conclusions of the study for the the objectives mentioned in the abstract as 'This paper studies how to design a distribution channel strategy and provides a product quality control strategy' are not very clear.<br>4. In this article, we can not see how consumer utility and consumer behavior affect product market demand.<br>5. In Mixed Channel, how does the product demand between the two channels affect each other?
6. PLOS authors have the option to publish the peer review history of their article (what does this	No

mean?). If published, this will include your full peer review and any attached files.<br><br>If you choose "no", your identity will remain anonymous but your review may still be made public.<br><br>**Do you want your identity to be public for this peer review?** For information about this choice, including consent withdrawal, please see our Privacy Policy.	
**Confidential to Editor**<br><br>1. Do you have any potential or perceived competing interests that may influence your review? Please review our Competing Interests policy and declare any potential interests that you feel the Editor should be aware of when considering your review. If you have no competing interests, please write "I have no competing interests."	I have no competing interests.
2. Did you receive any assistance in preparing this review (e.g. from a post-doc or graduate student)? If yes, please provide his/her name and title here. If you have chosen to reveal your identity to the authors in question 6 above, please include the name of the person who assisted you in the response to question 6 as well. (optional)	
3. If accepted, do you think this submission should be highlighted on the PLOS ONE website? PLOS ONE does not evaluate manuscripts based on perceived significance or readership. We aim to provide tools for readers to filter and evaluate our publications. (optional)	

More Reviewer Details

Cancel Save and Close
