# Supplementary material for: Supply chain product quality control strategy in three types of distribution channels
Source: PLoS One. 2020 Apr 22;15(4):e0231699. doi: 10.1371/journal.pone.0231699 (PMC7176101; doi:10.1371/journal.pone.0231699)
Supplement: Attachment — Submitted filename: Editor - initial review.docx [file pone.0231699.s001.docx]

**Editor's initial comments to the paper:**

**Supply Chain Product Quality Control Strategy in Three Types of Distribution Channels**

**T**he paper deploys a three-stage Stackelberg dynamic game analysis and constructs a product quality control strategy model for three types of distribution channels (direct channel, retail channel, and mixed channel) in a three-echelon supply chain, which is composed of one manufacturer, one retailer and the final customer. Furthermore, the paper studies how to design a distribution channel strategy and provides a product quality control strategy. Here, three types of distribution channels strategy in the context of how they influence a manufacturer’s product quality decision and prevention strategy, a retailer’s product pricing decision and quality inspection strategy, and the final customer’s product demand decision, are analyzed. The subject of this research is up-to-date and fundamentally interesting for scholars from the field of SCM and OR.

The paper, in general, roughly satisfies all rigor requirements that are demanded from Plos One. The red clue remains more or less consistent all over the paper, while the derivations of equations seem to be adequate.

However, the editor has detected some major issues based on his own perception, the review of the additional reviewer, and the Plos One criteria for the papers, which should be corrected prior to the further publishing process.

**Comments of the AE:**

1. It is not clear enough emphasized what the main contribution of the paper is, i.e., what has been done new, if compared with the work of the other researchers. Here, the borderline should be clearly highlighted, while the main contributions (novelties, originality) should be more explicitly given in the introduction and other places, where necessary.
2. Figure 1 should be improved in the sense of informative content meaning that the reader immediately understands the main point without even looking at the corresponding text.

**Comments regarding the Plos One criteria for the papers:**

There exists a certain doubt whether the paper meets PLOS ONE criteria for papers that describe new methods or software for applications. Specifically, these reports must meet the criteria of utility, validation, and availability, which are described in detail at <http://journals.plos.org/plosone/s/submission-guidelines#loc-methods-software-databases-and-tools>. On this basis, the AE warns the authors to reconsider these facts and give the rationale and/or explanation fortified with the facts, let be in the paper itself or in the answers to reviewers or cover letter.

**Comments of Reviewer #1**

Summary (see **reviewer 1 comments.pdf**):

Additional comments are available in the file: **reviewer 1 comments.pdf.**
