# Supplementary material for: Supply chain product quality control strategy in three types of distribution channels
Source: PLoS One. 2020 Apr 22;15(4):e0231699. doi: 10.1371/journal.pone.0231699 (PMC7176101; doi:10.1371/journal.pone.0231699)
Supplement: Attachment — Submitted filename: Response to Reviewers.doc [file pone.0231699.s003.doc]

Responses to Editors and Reviewers

Dear Professor Dejan Dragan and Reviewers,

Thank you very much for your suggestions and critical comments about our paper submitted to *PLOS ONE* (Manuscript ID: ***PONE-D-19-20228***). The revised title is “*Supply Chain Product Quality Control Strategy in Three Types of Distribution Channels*”.

We are also thankful to the reviewers for their critical reading and valuable comments on the manuscript. Those comments were very helpful for providing direction for our further studies. We have tried our best to revise our manuscript according to the comments. Attached, please find the revised version, which we would like to resubmit for your kind consideration. The main revised parts are marked in blue in the paper. The following is a detailed explanation how we have complied with the editor’s and reviewers’ suggestions.

**Responds to the editor’s comments:**

**Comment #1:**

The paper deploys a three-stage Stackelberg dynamic game analysis and constructs a product quality control strategy model for three types of distribution channels (direct channel, retail channel, and mixed channel) in a three-echelon supply chain, which is composed of one manufacturer, one retailer and the final customer. Furthermore, the paper studies how to design a distribution channel strategy and provides a product quality control strategy. Here, three types of distribution channels strategy in the context of how they influence a manufacturer’s product quality decision and prevention strategy, a retailer’s product pricing decision and quality inspection strategy, and the final customer’s product demand decision, are analyzed. The subject of this research is up-to-date and fundamentally interesting for scholars from the field of SCM and OR.

The paper, in general, roughly satisfies all rigor requirements that are demanded from Plos One. The red clue remains more or less consistent all over the paper, while the derivations of equations seem to be adequate.

**Response**: Thank you very much for the highly praises and the valuable suggestions. Our paper constructs a product quality control strategy model for three types of distribution channels (direct channel, retail channel and mixed channel) in a three-echelon supply chain which use a three-stage stackelberg dynamic game analysis, and then, analyzes three types of distribution channels strategy in the context of how they influence a manufacturer’s product quality decision and quality prevention strategy, a retailer’s product pricing decision and quality inspection strategy, and the final customer’s product demand decision. Most importantly, we conduct a numerical sample analysis that will prove the model’s effectiveness and indicate a specific application in practice.

**Comment #2:**

It is not clear enough emphasized what the main contribution of the paper is, i.e., what has been done new, if compared with the work of the other researchers. Here, the borderline should be clearly highlighted, while the main contributions (novelties, originality) should be more explicitly given in the introduction and other places, where necessary.

**Response**: Thank you very much for the valuable suggestions. We rewrite and emphasize the paper’s main contributions in every paragraph in “2 Related Literature”, just like: (1) Our paper differs from the existing literature by introducing the distribution channel demand elasticity ratio and investigating how to construct a product quality control strategy model and channel coordination in three types of distribution channels (direct channel, retail channel, and mixed channel) by providing a new perspective. (2) Our model contributes to the product quality control strategy research by constructing a distribution channel model in a three-echelon supply chain which is composed of one manufacturer, one retailer and the final customer based on a three-stage stackelberg dynamic game. Then, the model considers the manufacturer’s product quality decision and quality prevention strategy, the retailer’s product pricing decision and quality inspection strategy, and the final customer’s product demand decision. (3) we also establish a product quality control strategy model in three types of distribution channels to eliminate the influence of “channel conflict” and “free-riding behavior”, which will improve the manufacturer’s and retailer’s expected profits and the final customer’s consumer surplus.

**Comment #3:**

Figure 1 should be improved in the sense of informative content meaning that the reader immediately understands the main point without even looking at the corresponding text.

**Response**: Thank you very much for the valuable suggestions. We redraw the Figure 1 Three types of distribution channels decision system which improve in the sense of informative content meaning that the reader immediately understands the main point of our paper.

Figure 1 Three types of distribution channels decision system

**Responds to the reviewer’s comments:**

**Comment #1:**

The language of the article should be more concise and accurate.

**Response**: Thank you very much for the valuable suggestions. We made necessary revisions and language editing in the manuscript according to your suggestions, and the English has also been edited by Wiley English Language Editing Services.

**Comment #2:**

It is better to complement the differences between this study and the existing research literature.

**Response**: Thank you very much for the valuable suggestions. We rewrite and emphasize the paper’s main contributions in every paragraph in “2 Related Literature”, just like: (1) Our paper differs from the existing literature by introducing the distribution channel demand elasticity ratio and investigating how to construct a product quality control strategy model and channel coordination in three types of distribution channels (direct channel, retail channel, and mixed channel) by providing a new perspective. (2) Our model contributes to the product quality control strategy research by constructing a distribution channel model in a three-echelon supply chain which is composed of one manufacturer, one retailer and the final customer based on a three-stage stackelberg dynamic game. Then, the model considers the manufacturer’s product quality decision and quality prevention strategy, the retailer’s product pricing decision and quality inspection strategy, and the final customer’s product demand decision. (3) we also establish a product quality control strategy model in three types of distribution channels to eliminate the influence of “channel conflict” and “free-riding behavior”, which will improve the manufacturer’s and retailer’s expected profits and the final customer’s consumer surplus.

**Comment #3:**

The conclusions of the study for the objectives mentioned in the abstract as ‘This paper studies how to design a distribution channel strategy and provides a product quality control strategy’ are not very clear.

**Response**: Thank you very much for the valuable suggestions. With the changing in customer or consumer buying behavior, more and more companies are beginning to redesign or rebuild their distribution channel structure, Such as HP, Nike, Lenovo, in addition to focus on the traditional retail channel, have also opened up an internet channel(direct channel); Dell, MI has been focused on internet channel in the past, and now also began selling products in traditional retail channel; and Apple, Haier sell their products in the traditional retail channel and internet channel in the same time, which used a mixed channel structure. In our paper, we will construct a distribution channel strategy model in a three echelon supply chain that is composed of one manufacturer, one retailer and a final customer based on a three-stage stackelberg dynamic game. Furthermore, we will introduce the distribution channel demand elasticity ratio and investigate how to craft a product quality control strategy in three types of distribution channels (direct channel, retail channel, and mixed channel), Most important, we will determine the manufacturer’s product quality level, quality prevention effort level, wholesale price, direct sale price, and the retailer’s product quality inspection effort level and retail price, the manufacturer’s and retailer’s expected profits function, and the final customer’s consumer surplus.

**Comment #4:**

In this article, we can not see how consumer utility and consumer behavior affect product market demand.

**Response**: Thank you very much for the valuable suggestions. In “3 The Model and Assumption”, we describe that the final customer’s quality utility is$\theta q_{i}$, and$\theta$denotes the type of final customer, $q_{i}$is the manufacturer’s product quality level; furthermore, $i\in\{D,R,MC\}$denote the direct channel, the retail channel and the mixed channel respectively; then, we assume$\theta$～$U[a,b]$uniform distribution, and the corresponding final customer’s consumer surplus is$(\theta q_{i}-p_{i})$.

Therefore, in direct channel, we can describe the final customer’s consumer surplus as $CS^{D*}$=$\int_{a}^{b} (\theta q_{D}^{*}-p_{D}^{*})f(\theta)d\theta$=$(a+b)\alpha/3k\beta_{D}-4\alpha^{2}/9k\beta_{D}^{2}$ (14)

In retail channel, we can describe the final customer’s consumer surplus as

$CS^{R*}$=$\int_{a}^{b} (\theta q_{R}^{*}-p_{R}^{*})f(\theta)d\theta$=$(a+b)\alpha/3k\beta_{R}-5\alpha^{2}/9k\beta_{R}^{2}$ (30)

In mixed channel, we can describe the final customer’s consumer surplus as

$CS^{MC*}$=$\int_{a}^{(a+b)/2} (\theta q_{MC}^{*}-p_{D}^{MC*})f(\theta)d\theta$+$\int_{(a+b)/2}^{b} (\theta q_{MC}^{*}-p_{R}^{MC*})f(\theta)d\theta$

=$\frac{(a+b)(4-m)\alpha}{3k(\beta_{D}+\beta_{R})}-\frac{1}{2}[\frac{\alpha^{2}(4-m)^{2}}{6k(\beta_{D}+\beta_{R})^{2}}+\frac{\alpha^{2}(4-m)(3\beta_{D}+2\beta_{R})}{6k\beta_{D}\beta_{R}(\beta_{D}+\beta_{R})}]$ (52)

**Comment #5:**

In Mixed Channel, how does the product demand between the two channels affect each other?

**Response**: Thank you very much for the valuable suggestions. In our paper, we describe the mixed channel that the manufacturer sells their products in the traditional retail channel by the retailer, in the same time, the manufacturer sells their products to the final customer in direct channel (internet channel). The final customer’s product demand function will be$D_{i}(q_{i})=\alpha-\beta_{i}p_{i}/q_{i}$; $\alpha$denotes the market maximum demand, and$\beta_{i}$is the product demand price elasticity. Therefore, In mixed channel, the manufacturer and the retailer’s stackelberg “leader-follower” control model can be described as follows:

$Max\Pi_{M}^{MC}(q_{MC},\lambda_{m},w,P_{D})$=$(p_{D}-kq_{MC}^{2}/2)(\alpha-\beta_{D}p_{D}/q_{MC})(1-e^{-\lambda_{m}})$

+$(w-kq_{MC}^{2}/2)(\alpha-\beta_{R}p_{R}/q_{MC})(1-e^{-\lambda_{m}})$-$\eta_{m}\lambda_{m}$-$T$ (31)

*s.t.* $\left\{\lambda_{r},p_{R}\right\}=\arg Max\Pi_{R}^{MC}(\lambda_{r},p_{R})$

$Max\Pi_{R}^{MC}(\lambda_{r},p_{R})$=$(p_{R}-w)(\alpha-\beta_{R}p_{R}/q_{MC})(1-e^{-\lambda_{r}})-\eta_{r}\lambda_{r}+T$ (32)

The product demand in retail channel as $D_{R}(q_{MC})=(p_{R}-w)(\alpha-\beta_{R}p_{R}/q_{MC})$

The product demand in direct channel as $D_{D}(q_{MC})=(p_{D}-C_{D}(q_{MC}))(\alpha-\beta_{D}p_{D}/q_{MC})$

$q_{MC}$is the manufacturer’s product quality level in mixed channel, $\beta_{R}$is the product demand price elasticity in retail channel, $\beta_{D}$is the product demand price elasticity in direct channel, $w$ is the manufacturer’s wholesale price, $p_{R}$ is the retailer’s retail price,$p_{D}$ is the direct sale price in a direct channel, the manufacturer’s product quality cost function in direct channel is $C_{D}(q_{MC})=kq_{MC}^{2}/2$.

We have tried our best to improve the manuscript and made some substantial changes and necessary deletions according to the editors’ and reviewers’ comments. We earnestly appreciate the editors’ and reviewers’ professional work and hope that the corrections will make our manuscript suitable for publication in *PLOS ONE*. We are looking forward to receiving comments from reviewers in the future.

Once again, thank you very much for your valuable comments and suggestions.

Best wishes.

Yours sincerely,

Lilong Zhu	(*Correspondence*)

E-mail: zhulilong2008@126.com

Tel: + 86 13853193366, Fax: + 86 531 8618 2769

School of Management, Shandong University, Ji’nan Shandong, 250100, China

College of Business, Shandong Normal University, Ji’nan 250014, Shandong, China
