# Supplementary material for: Supply chain product quality control strategy in three types of distribution channels
Source: PLoS One. 2020 Apr 22;15(4):e0231699. doi: 10.1371/journal.pone.0231699 (PMC7176101; doi:10.1371/journal.pone.0231699)
Supplement: Attachment — Submitted filename: Response to Reviewers.doc [file pone.0231699.s004.doc]

Responses to Editors and Reviewers

Dear Professor Dejan Dragan and Reviewers,

Thank you very much for your suggestions and valuable comments about our paper submitted to *PLOS ONE* (Manuscript ID: ***PONE-D-19-20228R1***). The manuscript’s title is “*Supply Chain Product Quality Control Strategy in Three Types of Distribution Channels*”.

We are also thankful to the reviewers for their critical reading and valuable comments on the manuscript. Those comments were very helpful for providing direction for our further studies. We have tried our best to revise our manuscript according to the comments. Attached, please find the revised version, which we would like to resubmit for your kind consideration. The main revised parts are marked in *red* in the paper. The following is a detailed explanation how we have complied with the editor’s and reviewers’ suggestions.

**Responds to the editor’s comments:**

**Comment:**

Please include the following items when submitting your revised manuscript:

•	A rebuttal letter that responds to each point raised by the academic editor and reviewer(s). This letter should be uploaded as separate file and labeled 'Response to Reviewers'.

•	A marked-up copy of your manuscript that highlights changes made to the original version. This file should be uploaded as separate file and labeled 'Revised Manuscript with Track Changes'.

•	An unmarked version of your revised paper without tracked changes. This file should be uploaded as separate file and labeled 'Manuscript'.

**Response**: Thank you very much for the highly praises and the valuable suggestions for this manuscript. According to the editor’s and reviewers’ suggestions, we have completed all the revision which each point raised by the academic editor and reviewers, the file is labeled 'Response to Reviewers'. We have completed a marked-up copy of our manuscript that highlights changes made to the original version, the file is labeled 'Revised Manuscript with Track Changes'. We also have completed an unmarked version of our revised paper without tracked changes, the file is labeled 'Manuscript'.

**Comment #2:**

Please note while forming your response, if your article is accepted, you may have the opportunity to make the peer review history publicly available. The record will include editor decision letters (with reviews) and your responses to reviewer comments. If eligible, we will contact you to opt in or out.

**Response**: Yes, I would like to make the peer review history publicly available.

**Responds to the reviewer’s comments:**

**Comment #1:**

If the authors have adequately addressed your comments raised in a previous round of review and you feel that this manuscript is now acceptable for publication, you may indicate that here to bypass the “Comments to the Author” section, enter your conflict of interest statement in the “Confidential to Editor” section, and submit your "Accept" recommendation.

Reviewer #2: (No Response)

Reviewer #3: All comments have been addressed

**Response**: Thank you very much for the valuable suggestions. We have addressed and completed all the revised comments in the new 'Manuscript'.

**Comment #2:**

Is the manuscript technically sound, and do the data support the conclusions?

The manuscript must describe a technically sound piece of scientific research with data that supports the conclusions. Experiments must have been conducted rigorously, with appropriate controls, replication, and sample sizes. The conclusions must be drawn appropriately based on the data presented.

Reviewer #2: Yes

Reviewer #3: Yes

**Response**: Thank you very much for the valuable suggestions. In this manuscript, all the data support the conclusions.

**Comment #3:**

Has the statistical analysis been performed appropriately and rigorously?

Reviewer #2: Yes

Reviewer #3: Yes

**Response**: Thank you very much for the valuable suggestions.

**Comment #4:**

Have the authors made all data underlying the findings in their manuscript fully available?

Reviewer #2: Yes

Reviewer #3: Yes

**Response**: Thank you very much for the valuable suggestions.

**Comment #5:**

Is the manuscript presented in an intelligible fashion and written in standard English?

Reviewer #2: Yes

Reviewer #3: Yes

**Response**: Thank you very much for the valuable suggestions. We made necessary revisions and language editing in the manuscript according to your suggestions, and the English has also been edited by *Wiley English Language Editing Services*.

**Comment #6:**

Review Comments to the Author.

Reviewer #2: Really, the paper has been greatly improved as all comments provided by all other reviewers in the first round.

Reviewer #3: 1. It is a very nice thing that the new literature review part is added. But it seems to be to be too detailed. It will be better that the author just mentioned 2~3 related papers and on top of that, provide their own contributions. Moreover, those contributions should be more precise, instead of ". by providing a new perspective, the author need to be more specific about what exactly the new perspective is.

2. The model and assumption need to be more concise and concrete. For example, when the author mentioned the customer type follow a U[a,b], it needs more clarify about the value means and the types they are referring to. It will be better if the author check the model and assumption part one more time and provide additional explanation if necessary.

**Response to Reviewer #2**: Thank you very much for the valuable suggestions. We have revised and completed all comments provided by the editor and reviewers in the first round.

**Response to Reviewer #3**: Thank you very much for the valuable suggestions.

1. We have revised the literature review part, which is chiefly related to three streams of literature, it seems not to be too detailed, which is mentioned the related papers and on top of that, provide their own contributions. Moreover, those contributions should be more precise as *line 150-160.*

*In this paper, first of all, we will introduce the distribution channel demand elasticity ratio and investigating how to construct a product quality control strategy model and channel coordination in three types of distribution channels (direct channel, retail channel, and mixed channel); what’s more, we consider the manufacturer’s product quality decision and quality prevention strategy, the retailer’s product pricing decision and quality inspection strategy, and the final customer’s product demand decision in a three-echelon supply chain; above all, we also establish a product quality control strategy model in three types of distribution channels to eliminate the influence of “channel conflict” and “free-riding behavior”, which will improve the manufacturer’s and retailer’s expected profits and the final customer’s consumer surplus.*

2. We have revised model and assumption more concise and concrete in *line 174-l96*.

For example, The final customer’s quality utility is$\theta q_{i}$, and$\theta$denotes the type of final customer; then, we assume *Ɵ~U[a, b]* uniform distribution, i.e. *a* is the final customer lower limit of distribution quantity, *b* is the final customer upper limit of distribution quantity, and the corresponding final customer’s consumer surplus is$(\theta q_{i}-p_{i})$.

We have checked the model and assumption part one more time, and then, provided additional explanation more clearly and accurately.

We have tried our best to improve the manuscript and made some substantial changes and necessary deletions according to the editors’ and reviewers’ comments. We earnestly appreciate the editors’ and reviewers’ professional work and hope that the corrections will make our manuscript suitable for publication in *PLOS ONE*. We are looking forward to receiving comments from reviewers in the future.

Once again, thank you very much for your valuable comments and suggestions.

Best wishes.

Sincerely yours,

Lilong Zhu
